# Prescription of off-label and unlicensed medication for newborns hospitalized in the Intensive Care Unit

**DOI:** 10.1590/1984-0462/2024/42/2023023

**Published:** 2023-09-15

**Authors:** Laura Goedel dos Santos, Júlia Goedel dos Santos, Betine Moehlecke Iser, Kelser de Sousa Kock, Karla Dal Bó

**Affiliations:** a Universidade do Sul de Santa Catarina, Tubarão, SC, Brazil.; b Universidade do Planalto Catarinense, Lages, SC, Brazil.

**Keywords:** Off-label use, Unlicensed use, Drug prescriptions, Neonatal health, Neonatal intensive care, Drug legislation, Uso *off-label*, Uso sem licença, Prescrição de medicamentos, Saúde do lactente, Terapia intensiva neonatal, Legislação de medicamentos

## Abstract

**Objective:**

To analyze the prevalence of off-label and unlicensed prescriptions for a population of neonates admitted to the Neonatal Intensive Care Unit in a hospital in southern Santa Catarina.

**Methods:**

Observational study with a cross-sectional design. All neonates admitted to the Intensive Care Unit during the period from March 2020 to March 2021 were included. Data collection was performed through a questionnaire made by the authors and the classification of drugs based on the Electronic Drug Description (*Bulário Eletrônico*) of the Brazilian Health Regulatory Agency and Drug Dex-Micromedex.

**Results:**

Data from 296 neonates were evaluated. The prevalence was 50,7% for prescribing off-label medications and 37,2% for unlicensed medications. The use of drugs was higher in preterm neonates, with low birth weight, 1st minute Apgar between 6–8, 5th minute Apgar between 7–8, and in need of invasive procedures. The most used off-label drugs were ampicillin, gentamicin and fentanyl (92.6, 92.0 and 26.6%, respectively), whereas the most used unlicensed drugs were caffeine, phenobarbital and bromopride (78.1, 16.3 and 10.9%, respectively).

**Conclusions:**

This study showed a large percentage of prescriptions made in the off-label (50.7%) and unlicensed (37.2%) form in the Neonatal Intensive Care Unit, corroborating the worrying world scenario. The most exposed neonates were precisely the most vulnerable ones and, among the most commonly prescribed medications, ampicillin and gentamicin stood out in off-label form and caffeine in unlicensed form.

## RESUMO

**Objetivo:**

Analisar a prevalência de prescrições *off-label* e não licenciadas uma população de neonatos internados na Unidade de Terapia Intensiva Neonatal em um hospital ao sul de Santa Catarina.

**Métodos:**

Estudo observacional com delineamento transversal. Foram incluídos todos os neonatos admitidos na Unidade de Terapia Intensiva durante o período de março de 2020 a março de 2021. A coleta de dados foi realizada a partir de questionário elaborado pelos autores, e a classificação dos medicamentos, com base no Bulário Eletrônico da Agência Nacional de Vigilância Sanitária e no *Drug Dex-Micromedex*.

**Resultados:**

Foram avaliados dados de 296 neonatos. A prevalência foi de 50,7% para prescrição de medicações *off-label* e 37,2% para medicações não licenciadas. O uso dos fármacos foi maior em neonatos pré-termo, com baixo peso ao nascer, Apgar de 1^o^ minuto entre 6–8, Apgar de 5^o^ minuto entre 7–8, e com necessidade de procedimentos invasivos. Os fármacos *off-label* mais utilizados foram a ampicilina, gentamicina e fentanil (92,6, 92 e 26,6%, respectivamente), já os fármacos não licenciados mais utilizados foram a cafeína, fenobarbital e bromoprida (78,1, 16,3 e 10,9%, respectivamente).

**Conclusões:**

O estudo demonstrou grande porcentagem de prescrições realizadas de forma *off-label* (50,7%) e não licenciada (37,2%) na Unidade de Terapia Intensiva Neonatal de análise, corroborando o preocupante cenário mundial. Os neonatos mais expostos foram justamente aqueles mais vulneráveis e, entre as medicações mais utilizadas, destacam-se a ampicilina e a gentamicina de modo *off-label* e a cafeína de modo não licenciado.

## INTRODUCTION

Off-label (OL) are defined as those drugs that, despite having regulatory approval for use in the country, are prescribed differently from what is indicated on the package insert — whether in relation to posology, indication, age group, administration interval, and/or form of administration.^1^ Unlicensed drugs (UL) have three main definitions:

- drugs without authorization to be marketed in the country,
- drugs that do not have an appropriate formulation on the market, and
- drugs with preparation and use of extemporaneous formulas, conceived from the grinding of tablets, dilution of oral liquids, or opening of capsules.^1,2^

The prescription of OL and UL drugs can be considered a common practice in the pediatric area, both in hospitals and outpatient clinics.^3,4^ This reality is conditioned by the scarcity of regulations for medications in the pediatric population, the disparity in scientific studies on the action of drugs in pediatrics, and the lack of knowledge of some professionals on the subject.^5^ If drug prescription is complex in children, the proper use of medications in the neonatal period — especially in the need for admission to the Intensive Care Unit — is even more intimidating.^6^

The neonatal period comprises the first 28 days of a baby's life and is considered a phase of vulnerability to the child health owing to biological, environmental, social, and cultural factors.^7^ In addition to the risks naturally expected between the first and 28th day of life, due to the physiological peculiarities of this age group, newborns (NBs) who, for some reason, require intensive care and multiple pharmaceutical treatments are even more exposed to negative outcomes — among them the increased risk of adverse drug reactions and prescription errors.^8,9^

Although the administration of OL and UL drugs promotes risks higher than those already defined for regulated drugs,^10^ their use is widespread in the management of neonates admitted to Neonatal Intensive Care Units (NICUs). OL prescriptions account for 38–99.5% of all drugs used in NICUs^2,11–14^ while the ULs have a prevalence of use ranging from 1.9–24%.^2,12,14–16^ The percentage variation is due to different study designs and clinical settings, as well as the adopted definition of OL and UL drugs.^14^ Because of the heterogeneity of results, there is still no clear definition of the prevalence of these prescriptions in practice, nor is known the magnitude of the risks to which neonates are actually exposed. The small number of clinical trials conducted with this population, especially due to ethical and methodological factors, is responsible for the low evidence of safety of drug prescriptions.^17^

The high prevalence of OL and UL prescriptions is also a reality in our country, corroborating worldwide trends. Brazil, despite having a regulatory agency for drug registration — Brazilian Health Regulatory Agency (ANVISA) — still does not have specific regulations for the neonatal population, making it impossible to reduce the prevalence of these prescriptions. The use of these products, even “globalized”, lacks scientific contributions that allow an adequate understanding of the true risks and benefits of this practice.

The present study sought to analyze the prevalence of OL and UL prescriptions in neonates admitted to the NICU of a regional hospital in southern Santa Catarina, Brazil, besides performing a secondary analysis of the epidemiological profile of the newborns who received these medications. We believe that this research can encourage future development of appropriate drugs, thereby reducing damage to the neonatal population, especially since newborns are already subject to risks inherent to the severity of the pathologies that require hospitalization and treatment at such an early stage of their existence.

## METHOD

This is an observational study with cross-sectional design, through research in secondary databases. The study population was composed of neonates admitted to the NICU of the Hospital Nossa Senhora da Conceição (HNSC), from March, 2020 to March, 2021, in the city of Tubarão, Santa Catarina state, Brazil.

The list was provided by the Information Technology Sector of the HNSC and all neonates admitted to the NICU of this hospital were included, being initially 397 neonates admitted to the sector. The defined exclusion criteria were incomplete or lost socio-demographic data, with 101 newborns considered as losses.

Data collection was initiated after approval by the Research Ethics Committee of the Universidade do Sul de Santa Catarina, under opinion 5,226,515 on February 7, 2022, respecting the precepts of resolution 466/12 of the National Health Council. The data were documented in a Data Collection Protocol, previously prepared by the authors, and were then computerized.

The protocol was composed of three groups of variables: sociodemographic (gestational age, birth weight, route of delivery, and Apgar score on the 1st and 5th minutes), prescription-related (use of OL and UL medication, total number of OL and UL prescriptions, main OL and UL medications prescribed), and clinic-related (diagnosis at hospitalization, need for invasive procedures, invasive procedures used, and outcome).

The invasive procedures evaluated in this study were: urinary catheter, venous dissection, umbilical catheter, parenteral nutrition, mechanical ventilation, peripheral venous access, arterial gasometry and peripherally inserted central catheter.

The variables related to prescriptions were defined based on the classification of drugs as OL and/or UL for the analyzed population. This definition was performed according to national data by ANVISA's Electronic Drug Description (*Bulário Eletrônico*) and international data by Drug Dex-Micromedex, allowing the comparison of results with international institutions.

After collection, data were tabulated in Microsoft Excel 2016 spreadsheet and statistical analysis was performed in Statistical Package for the Social Sciences (SPSS) software, version 21.0. Quantitative variables were described using measures of central tendency and data dispersion, while qualitative variables were described using absolute and percentage frequency. Differences in proportions were tested by Pearson's chi-square (X^2^) test and differences in means, by Student's *t*-test or non-parametric equivalents, according to data suitability. The statistical significance level adopted was 5% (p-value<0.05).

## RESULTS

In this study, 296 records of neonates submitted to care in the NICU, at Hospital Nossa Senhora da Conceição, during a one-year period were evaluated. The mean gestational age was 34.2±4.0 weeks, and the mean birth weight was 2,348.1±942.0 grams. The route of delivery was cesarean section in 216 births (72.9%) and the Apgar score represented good vitality (>7 points) at the 1st minute in 227 (76.6%) newborns and at the 5th minute in 272 (91.8%). The main admission diagnoses were respiratory distress (82.9%), prematurity (67.6%), and risk of early sepsis (39.5%); other less common were congenital pneumonia (4.7%), meconium aspiration syndrome (6, 2%), and congenital heart disease (1%). The need for invasive procedures was reported in 226 (76.3%) neonates.

The prevalence of OL prescriptions in the study population was 50.7%, while the prevalence of UL prescriptions was 37.2%. The use of OL and UL medications was notably higher in preterm neonates with low birth weight, 1st and 5th minute Apgar scores between 6–8, and with need for invasive procedures (Table 1). The main route of delivery was cesarean section, performed in 68.7% of all newborns who used OL medication and 70% of those who used UL medications.

**Table 1:** Clinical-demographic profile of the 296 neonates who received off-label or unlicensed medications during hospitalization in the Neonatal Intensive Care Unit of the Hospital Nossa Senhora da Conceição, in Tubarão (SC), between March 2020 and March 2021.

		Use of off-label medication		p-value	Use of unlicensed medication		p-value
		Yes	No		Yes	No	
GA (weeks)		32.7±4.6	35.7±2.5	<0.001	31.3±4.2	35.8±2.8	<0.001
Weight (g)		2021±969	2684±785	<0.001	1717±844	2721±787	<0.001
Apgar 1^st^ min.		6.7±2.5	7.7±1.7	<0.001	6.8±2.3	7.4 ±2.1	0.02
Apgar 5^th^ min		8.2±1.8	8.9±1.1	<0.001	8.2±1.6	8.7(±1.5	0.009
Invasive procedures						
	Yes	150 (100%)	76 (52.1%)	<0.001	106 (96.4%)	120 (64.5%)	<0.001
	No	0 (0%)	70 (47.9%)	<0.001	4 (3.6%)	66 (35.5%)	<0.001

Values in mean ± standard deviation or in number (%); GA: gestational age

**Figure 1:**
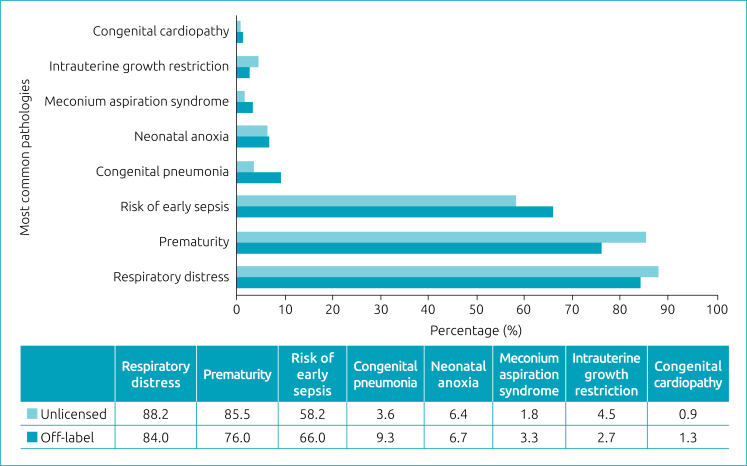
Main clinical conditions associated with admission to the Neonatal Intensive Care Unit and prescription of off-label or unlicensed medications at Hospital Nossa Senhora da Conceição, in Tubarão (SC), between March 2020 and March 2021.

The main pathologies at diagnosis associated with the use of OL and UL medications for treatment within the NICU analyzed were respiratory distress, prematurity and risk of early sepsis (Figure 1). Other less prevalent pathologies, representing less than 1% of the total, were achondroplasia, atresia of the choanae, atresia of the esophagus, cyanosis of the lower limbs, convulsive crisis, severe dehydration, severe jaundice, acute respiratory failure, cleft lip, cystic adenomatous malformation of the lung, congenital syphilis, omphalocele, and others.

**Figure 2:**
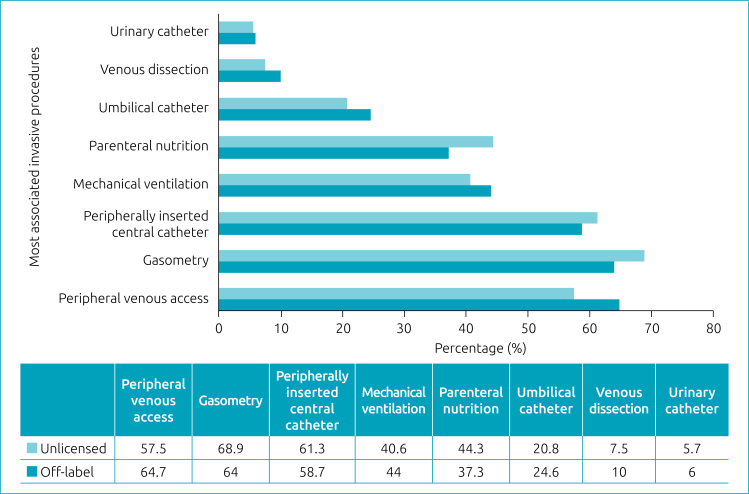
Main invasive procedures associated with admission to the Neonatal Intensive Care Unit and prescription of off-label or unlicensed medications at Hospital Nossa Senhora da Conceição, in Tubarão (SC), between March 2020 and March 2021.

Concerning the invasive procedures most commonly used in newborns admitted to the NICU who used OL and UL medications, we can mention mainly the peripherally inserted central venous catheter, arterial gasometry and peripheral venous access, as shown in Figure 2. Some invasive procedures were used in minimal prevalence: intrahepatic catheter and ventricular-atrial shunt.

Of the 296 neonates evaluated in the NICU during this period, 238 used between 0–5 medications and 58 used between 6–19 medications, approved or not. Of the total number of neonates who received OL prescriptions, 120 used between 1–3 OL medications, and the remaining 30 used between 4–8 medications in this category. Regarding the total number of neonates who received UL prescriptions, 96 of them used only one medication from this class and 14 used between 2–3 UL medications.

The most commonly prescribed OL drugs for the neonates were ampicillin, gentamicin, fentanyl, and vancomycin, and the most commonly UL drugs were caffeine, phenobarbital, and bromopride (Table 2).

**Table 2:** Frequency of off-label and unlicensed medications most used in the 296 neonates admitted to the Neonatal Intensive Care Unit of the Hospital Nossa Senhora da Conceição, in Tubarão (SC), between 2020 and 2021.

Off-label use		
Medication	Prescriptions (n)	%
Ampicillin	139	92.6
Gentamicin	138	92.0
Fentanyl	40	26.6
Vancomycin	32	21.3
Amikacin	20	13.3
Cefepime	13	8.6
Fluconazole	9	6.0
Meropenem	8	5.3
Metronidazole	7	4.6
**Unlicensed use**		
**Medication**	**Prescriptions (n)**	**%**
Caffeine	86	78.1
Phenobarbital	18	16.3
Bromopride	12	10.9
Dipyrone	6	5.4
Spironolactone	1	0.9
Captopril	1	0.9

The outcome of the study neonates was classified as hospital discharge and/or transference or death. Of the total 296 neonates analyzed, 22 (7.4%) died during hospitalization. Considering the newborns who used OL medications, the proportion of deaths was 14%, while among those who did not use OL medications, was only 0.7% (p<0.001). Among newborns who received UL medications, 11.8% died, and among those who did not receive, 4.8% died (p=0.027). The mean number of OL medications used in the neonates who did not survive was 3.9±1.9 drugs, while the mean number of UL medications used in this group was 1.2±0.4. The negative outcome was more prevalent in neonates who used more than five medications, regardless of category, representing 72.7% of deaths recorded in this period, and the mean total number of medications in this group was 9.0±5.2 drugs.

## DISCUSSION

The high prevalence of OL (50.7%) and UL (37.2%) prescriptions used in neonates demonstrated in this study corroborates some previously published national and international data. In a cohort study conducted in Natal (RN), 49.3% of neonates received OL prescriptions and 24.6% received UL prescriptions during hospitalization.^18^ In a Spanish cohort study, it was found that 57.1% of neonates received OL medications, while 32.1% received at least one UL medication.^19^

Demonstrating even higher prevalence when only the premature population was analyzed, a recent noncompeting cohort study conducted in Vitória da Conquista (BA), found a prevalence of 79% for OL use and 55.5% for UL use in infants born at less than 37 weeks gestational age.^20^ Another study, cited above, showed a 100% frequency of OL use of medications in extreme preterm (<28 weeks) and very preterm (between 28–31 weeks) infants.^14^ This data confirms the most prevalent mean age for use of these medications in the present study.

A cross-sectional study conducted in the NICU of an Italian hospital showed that 93.4% of neonates received OL prescriptions and 46.7% received UL.^21^ Another analysis, this time in Indian, showed that only 12.3% of prescriptions were used in OL form in the NICU.^22^ The great divergence between the data may be due to the different standardization of medications, based on the Drug Regulatory Agencies of each country — in Italy, the European Medicines Agency and, in India, the British Regulatory Agency.

In addition to the classifications according to national regulations, different definitions can be used when referring to OL and UL medications. It is even possible to group them into a large group defined only as OL, as performed in the articles cited below. A cross-sectional study carried out in a high-risk maternity hospital in Aracaju (SE) showed that 98.1% of neonates used OL therapy — including drugs not licensed by ANVISA.^5^ Another study, a six-month retrospective cohort, also in Brazil, showed that 95.6% of prescriptions in the NICU were OL.^22^ The difference in nomenclature and classification of the drugs hinders the comparison of this article with some other previously published studies.

Regarding the epidemiological clinical profile, as in this study, some authors have already demonstrated that the newborns that most received OL and UL prescriptions were preterm infants, with low birth weight, and 1st and 5th minute Apgar scores between 6–8. In a retrospective Brazilian study, unapproved prescriptions were notably higher (100% OL and 20.5% UL) in neonates with a gestational age <31 weeks, with a mean 1st minute Apgar score of 5.9 (standard deviation SD±2.0), mean 5th minute Apgar score of 7.9 (SD±1.3), and mean birth weight of 1,078.4g (SD±268.6).^13^ Furthermore, a Brazilian cohort study, also non-competing, showed an association of OL prescriptions with very preterm (28–31 weeks), extreme preterm (<28 weeks), low birth weight (<2,500 g), and 5th minute Apgar score <7. For UL use, the association was greater in very preterm infants, with birth weight between 1,000–1,500 g, and 5th minute Apgar score <7.^20^

The main diagnoses performed in the NICU that required the use of unapproved or different treatment from the regulations were precisely those most prevalent in the sector. A Brazilian cross-sectional study showed that respiratory syndrome and sepsis accounted for 56.9% of the diagnoses of newborns admitted to the NICU.^18^ Another study showed that prematurity was responsible for 48% of the admissions, while respiratory dysfunction was responsible for 18.9%.^23^ This article, based on secondary data collection, considered only the diagnosis that led to admission of the newborn to the NICU. One of its limitations, therefore, is the fact that it cannot include relevant pathologies that may have emerged during continued care or coexisted with the main pathology — with delayed diagnosis — since they may have influenced the definition of treatment.

The profile of neonates who required invasive procedures during hospitalization corresponds, almost in totality, with the profile of neonates who used the OL and UL medications (96.4% of those who used UL and 100% of those who used OL; p<0.001 in both) — a finding that confirms the exposure of the most fragile infants to the less studied drugs. The invasive procedures most associated with the concomitant use of the studied medications were peripheral venous access, arterial gasometry, and peripherally inserted central catheter.

In the present study, the most prescribed OL drugs were ampicillin, gentamicin, and fentanyl. Fentanyl, classified as an opioid, ranked 3rd among the OL drugs most prescribed in the hospital's NICU, and is widely used for neonatal pain control, although it is only allowed for pediatric use above 2 years of age.^24^ The drug belongs to the highest alert group, because if administered incorrectly it can culminate in serious outcomes.^25^ Ampicillin and gentamicin, in turn, are anti-infectives for systemic use and were the main drugs prescribed, accounting for 92.6 and 92% of neonates receiving OL medications, respectively. The percentage difference may have occurred because of an error in data collection — since the two medications are used in association in the NICU for the treatment of early neonatal sepsis — or due to the isolated use of ampicillin in one neonate.

Ampicillin, the main OL medication prescribed in this study, is only approved for pediatrics over 1 year of age. However, it is indicated by the guidelines for neonatal use, both for prophylaxis and treatment.^26^ Gentamicin, in turn, despite being approved for use in newborns, must be prescribed at 8- or 12-hour intervals according to ANVISA.^27^ In the reality of NICUs, the drug is prescribed every 24, 36 or 48 hours, according to the indication of neonatal protocols.^26^ A Brazilian study also demonstrated the main prevalence of amikacin use, but did not consider gentamicin as OL, despite being administered differently from the package insert in relation to the administration time between doses.^5^ Studies in other centers confirmed the class of anti-infectives as the main OL prescribed, but there were differences among the most prevalent antibiotics: amikacin, tobramycin, meropenem, cefotaxime and vancomycin, that were cited in different results.^11,12,28^

Regarding the most prescribed UL drugs, caffeine stood out in prevalence. Caffeine citrate, despite not being regulated by ANVISA, is used for the prevention and treatment of primary apnea of prematurity, acting on the central nervous system.^29^ In a survey carried out in Spain,^16^ caffeine, although being the most prescribed UL drug, had a prevalence of 33.3% — while in the present study it was responsible for 78.1% of the UL prescriptions. Another research, conducted in Bahia, had a prevalence of caffeine use of 75.5% among neonates submitted to UL drugs, which corroborates the prevalence found in this article.^19^ Other UL drugs prescribed, but in smaller amounts, were intravenous phenobarbital — a barbiturate allowed only orally by ANVISA — and bromopride — an antiemetic contraindicated for infants below one year of age.

Among newborns who used OL and/or UL medication, 14 and 11.8% respectively, died. The negative outcome was more prevalent in neonates who required more than five medications, regardless of drug class. In a Korean study,^29^ the mean number of OL and UL medications used was also higher in neonates who did not survive; however, it was not possible to directly associate the two variables. Although there is statistical significance between the number of medications used and the negative outcome, it is not possible to determine a cause-consequence relationship, since disease severity is related to the number of prescriptions received and also to the cause of death.

In conclusion, the current study demonstrated the high percentage of prescriptions made of OL (50.7%) or UL (37.2%) drugs in the NICU analyzed. The most vulnerable neonates to these prescriptions were those with low birth weight, born before 37 weeks of gestational age, with Apgar score between 6–8 and in need of invasive procedures. Among the most commonly used medications were ampicillin and gentamicin in an OL form and caffeine in UL. The results show a worrisome scenario, in agreement with worldwide data, which makes it essential to carry out further studies in the area, thus seeking to achieve greater safety and quality drug therapy in neonatology. In view of the ethical difficulty of performing clinical trials in this population, retrospective studies are of great value, since they can be used by drug regulatory agencies, whether national or not, to review drug package inserts.

## Data Availability

The database that originated the article is available with the corresponding author.
